# Supplementary material for: Recyclable Thin‐Film Soft Electronics for Smart Packaging and E‐Skins
Source: Adv Sci (Weinh). 2023 Jul 12;10(26):2301673. doi: 10.1002/advs.202301673 (PMC10502858; doi:10.1002/advs.202301673)
Supplement: Supplementary file 1 — Supporting Information [file ADVS-10-2301673-s002.pdf]

ADVANCED SCIENCE
Open Access

Supporting Information

for *Adv. Sci.*, DOI 10.1002/advs.202301673

Recyclable Thin-Film Soft Electronics for Smart Packaging and E-Skins

Manuel Reis Carneiro, Aníbal T. de Almeida, Mahmoud Tavakoli and Carmel Majidi**

Supporting Information

Recyclable thin-film soft electronics for smart packaging and e-skins

Manuel Reis Carneiro, Aníbal T. de Almeida, Mahmoud Tavakoli and Carmel Majidi**

M. Reis Carneiro, Professor C. Majidi*

Soft Machines Lab, Department of Mechanical Engineering, Carnegie Mellon University, Pittsburgh, Pennsylvania 15213, United States

E-mail: cmajidi@andrew.cmu.edu

M. Reis Carneiro, Professor Aníbal T. de Almeida, Professor M. Tavakoli*

Institute of Systems and Robotics, Department of Electrical and Computer Engineering, University of Coimbra, Coimbra 3030-290, Portugal

E-mail: mahmoud@isr.uc.pt

Table S1. Comparison between the contributions of this work and other works in the field, in terms of ink formulation, decomposition process, electrical conductivity, stretchability, usability for digital printing of circuits, and applications.

Figure S1. 0402-sized resistors and LEDs (1.0 x 0.5 mm package dimensions and 0.5 mm pad separation) bonded to a set of printed lines with 200 um width.

Figure S2. Influence of silver flake and liquid metal concentration in the inks' conductivity at 0% strain.

Figure S3. Variation of the viscosity of four Ag-WPU inks as a function of shear rate. The samples were stored at room temperature between 0 and 35 days.

Figure S4. Estimated conductivity of Ag-WPU samples upon stretching. The material was assumed to be incompressible.

Figure S5. Strain vs resistance curve for Ag-WPU traces printed over a thermoplastic polyurethane (TPU) substrate (nine samples from distinct ink batches).

Figure S6. SEM image of Ag-WPU surface

Figure S7. SEM image of Ag-WPU surface

Figure S8. Strain-Stress curves until break for printed ink trace (green) and printed trace with integrated SMD resistor (blue).

Figure S9. Printed circuit with integrated SMD components separated in its fundamental elements: conductive ink, TPU substrate, and rigid components.

Figure S10. Ag-rich powder after IPA washing. Large PU aggregates can be observed (red highlights).

Figure S11. Ag-rich powder after removal of large PU chunks (left) and after being transferred to a glass vial (right).

Figure S12. Microscope images of the surface of printed traces from pristine Ag-WPU ink (left) and recycled Ag-WPU ink (right). For the image on the right, a rough surface can be observed, originating from solidified PU residue trapped in the print.

Figure S13. Comparison between Strain vs resistance curve for pristine Ag-WPU traces and traces printed from recycled Ag-WPU.

Figure S14. Strain-stress curves for pristine TPU film and the same TPU film after undergoing the IPA recycling process.

Figure S15. Estimated conductivity of Ag-EGaIn-WPU samples upon stretching. The material was assumed to be incompressible.

Figure S16. 50 stretching/releasing cycle test of the Ag-WPU and Ag-EGaIn-WPU inks (ε = 10%). The insets on the bottom plots show 5 cycles in the middle of the test.

Figure S17. A: Microscope images of the surface of printed traces from Ag-EGaIn-WPU. A rough surface can be observed, originating from EGaIn droplets fully encapsulated in polyurethane **B:** DIW printed grids using the Ag-EGaIn-WPU ink with 1.37 mm and 812 um line spacing. A 250 um diameter nozzle was used.

Figure S18. SEM image and EDS analysis of Ag-EGaIn-WPU sample cross section. A clear separation between Ag and Ga can be observed, while In is present all over the sample

Figure S19. SEM image and EDS analysis of Ag-EGaIn-WPU sample cross section. A clear separation between Ag and Ga can be observed, while In is present all over the sample

Figure S20. SEM image and EDS analysis of Ag-EGaIn-WPU sample cross section. The formation of intermetallic Ag-In microparticles is observed. These can be distinguished from silver flakes thanks to their spherical shape

Figure S21. SEM image and EDS analysis of Ag-EGaIn-WPU sample cross section. The formation of intermetallic Ag-In microparticles is observed. These can be distinguished from silver flakes thanks to their spherical shape

Figure S22. Temperature plots from data acquired by the printed smart label.

Figure S23. Dynamic time warping fitting between the signals for two repetitions of a smiling gesture.

Figure S24. Dynamic time warping fitting between the signals for two repetitions of a jaw clenching gesture.

Figure S25. Dynamic time warping fitting between the signals for two repetitions of an eye blinking gesture.

Figure S26. Dynamic time warping fitting between the signals for two repetitions of an eyebrow flashing gesture.

Figure S27. Euclidean distance between gesture pairs of the two repetitions, obtained from Dynamic Time Warping of the electrophysiological signals.

Figure S28. Electrode-skin impedance (1 Hz-500 kHz) for printed Ag-WPU electrodes and medical-grade Ag/AgCl electrodes.

Figure S29. Steps for synthesis of Ag-EGaIn-WPU conductive ink.

Figure S30. Example of the printed traces over FR1 substrate to measure ink conductivity.

Figure S31. Die C, ASTM D412 dogbone with printed trace for tensile testing.

Figure S32. Die C, ASTM D412 dogbone with printed trace and integrated SMD resistor for tensile testing.

Figure S33. Example of the printed traces over FR1 substrate using recycled Ag-WPU ink. Rough surface texture arises from the presence of cured polyurethane particles in the recovered Ag-rich powder that become encapsulated in the new print.

Figure S34. Recyclable smart label circuit.

Figure S35. Setup for thermistor calibration consisting of a hotplate and digital thermometer.

Figure S36. Calibration curve for the thermistor-based temperature-monitoring electronic patch.

Figure S37. High level block diagram of the developed electrophysiology recording system.

Video S1. Ultraflexible printed circuit that is deformed (bent and crumpled) without losing functionality.

Video S2. Printed circuit with integrated SMDs being stretched and crumpled without delamination of the rigid components or the conductive traces from the soft substrate.

Video S3. Temperature data transfer between the printed smart package and a smartphone through Bluetooth low energy.

Video S4. Change in LED color in the smart label when the package is stored for more than one hour at temperatures higher than 5 ºC.

Video S5. Direct ink writing of soft circuits using the proposed Ag-WPU conductive ink.

Table S1. Comparison between the contributions of this work compared to other works in the field, in terms of ink formulation, decomposition process, electrical conductivity, stretchability, usability for digital printing of circuits, and applications.

Recyclable printable conductors

work	Ink composition	Liquid medium	Decomposition process	Conductivity	Stretchability	Digital Printing	Applications	Other
This work	Ag + WPU	water	Dissolution in IPA	1.6×10^5 S/m	Up to **200 %** (with the inclusion of LM which increases recycling complexity)	Yes	Fully integrated soft circuits with discrete SMD components<br><br>Electronic patches for health monitoring (temperature + biopotentials)<br><br>Smart labels for recyclable packages	A method to use the ink for 'soft soldering' of rigid microelectronic components is as well shown<br><br>Long shelf life at room temperature before printing
[1]	Ag + EGaIn + SIS	Toluene	Toluene vapor, IPA, Acetone, leaching & electrowinning	2.1×10^5 S/m	1700 %	Yes	Fully integrated soft circuits with discrete SMD components<br><br>Electronic patches for health monitoring	
[2]	EGaIn	-	Substrate is dissolved	1.5×10^6 S/m	150 %	No	Strain sensor with integrated smd components	
[3]	PVA-LM	water	Dissolution in NaOH	1.3×10^5 S/m	20 %	Yes	Strain + pressure sensors	Conduction must be 'activated ' by applying pressure
[4]	Tin solder	-	Remelting of the tin	Bulk metal	X	Yes	Blinking LEDs with discrete SMD components	Non-stretchable nor flexible
[5]	polycaprolactone + Ag + enzyme/protectant nanoclusters		Warm water	2.1×10^4 S/m	80 %	Yes	Strain sensor	

Some recent works have showed recyclable circuits, as shown in Table S1. For instance the work shown in reference [5] proposes a printable conductor with straightforward decomposition in warm water but nevertheless with limited stretchability (80%) and relatively low conductivity (2.1 x 10^4 S/m), as well as no demonstration of direct integration of microchips. Using fully metallic conductors, reference [4] introduces recyclability in rigid circuits made of tin-based conductive tracks that can be melted and reused, while [2] discloses stretchable LM-based stencil printed circuits used for strain sensing. An interesting contribution to the fields of ecological and recyclable electronics is shown in [3] where a PVA-LM mixture in a water medium can be digitally printed as mechanical deformation sensors yet this formulation presents limited stretchability (20%), doesn't prove suitable for microchip integration and the conductive patterns must be activated by an externally applied pressure thus adding another step to the fabrication process. More recently, [1] proposes a variety of highly promising methods for fabricating and recycling highly stretchable fully integrated circuits. Nevertheless, on the one hand the inks used all contain toluene as solvent (which entails environmental concerns) and as well the proposed recycling method requires cumbersome steps such as 'electrowinning' and 'leaching', while also using solvents such as acetone and toluene in the process. Hereupon, having in mind that a printed circuit without microchips would be of limited use, and complex multi-step recycling may prove itself ineffective in terms of cost and scalability, this work represents a striking advance compared to past efforts with recyclable electronics that are soft, stretchable, and printable, none were able to demonstrate a combination of high resolution digital printing, microchip integration, strain tolerance, and simple, efficient and ecological recycling, being this work the first to combine all of these factors.

Table References:

[1] Tavakoli, M. et al. 3R Electronics: Scalable Fabrication of Resilient, Repairable, and Recyclable Soft ‐ Matter Electronics. Advanced Materials vol. 34 2203266 (2022).

[2] Shi, C. et al. Stretchable, Rehealable, Recyclable, and Reconfigurable Integrated Strain Sensor for Joint Motion and Respiration Monitoring. Research vol. 2021 (2021).

[3] Xu, J. et al. Printable and Recyclable Conductive Ink Based on a Liquid Metal with Excellent Surface Wettability for Flexible Electronics. ACS Applied Materials & Interfaces vol. 13 7443–7452 (2021).

[4] Dou, Y., Luo, J., Qi, L., Lian, H. & Huang, J. Drop-on-demand printing of recyclable circuits by partially embedding molten metal droplets in plastic substrates. Journal of Materials Processing Technology vol. 297 117268 (2021).

[5] Kwon, J. et al. Conductive Ink with Circular Life Cycle for Printed Electronics. Advanced Materials vol. 34 2202177 (2022).

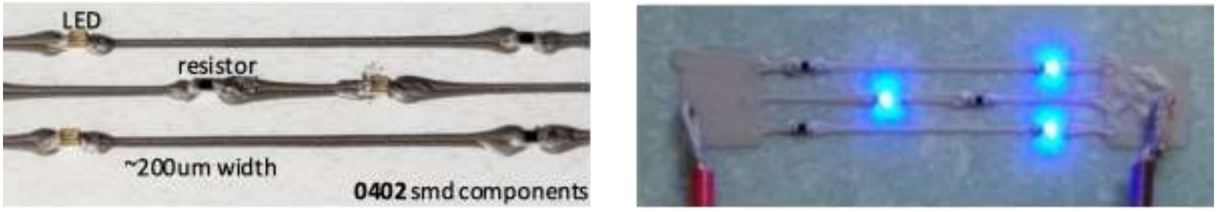

Figure S1. 0402-sized resistors and LEDs (1.0 x 0.5 mm package dimensions and 0.5 mm pad separation) bonded to a set of printed lines with 200 um width.

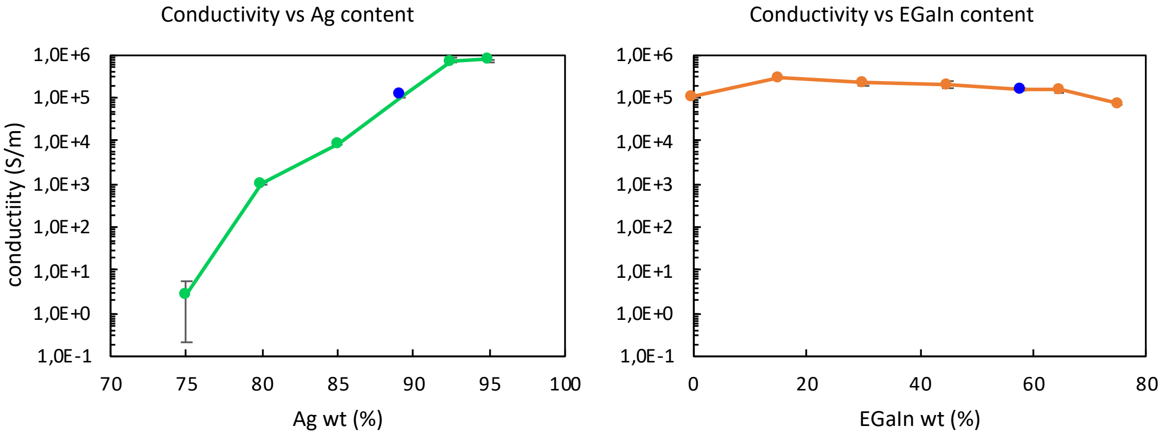

Figure S2. Influence of silver flake and liquid metal concentration in the inks' conductivity at 0% strain.

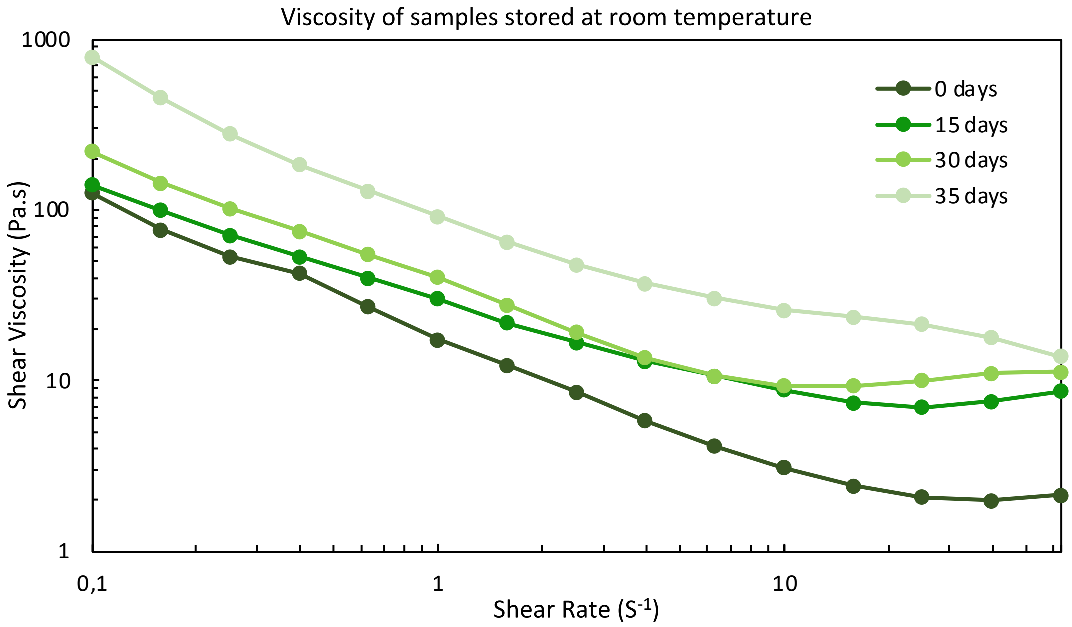

Figure S3. Variation of the viscosity of four Ag-WPU inks as a function of shear rate. The samples were stored at room temperature between 0 and 35 days.

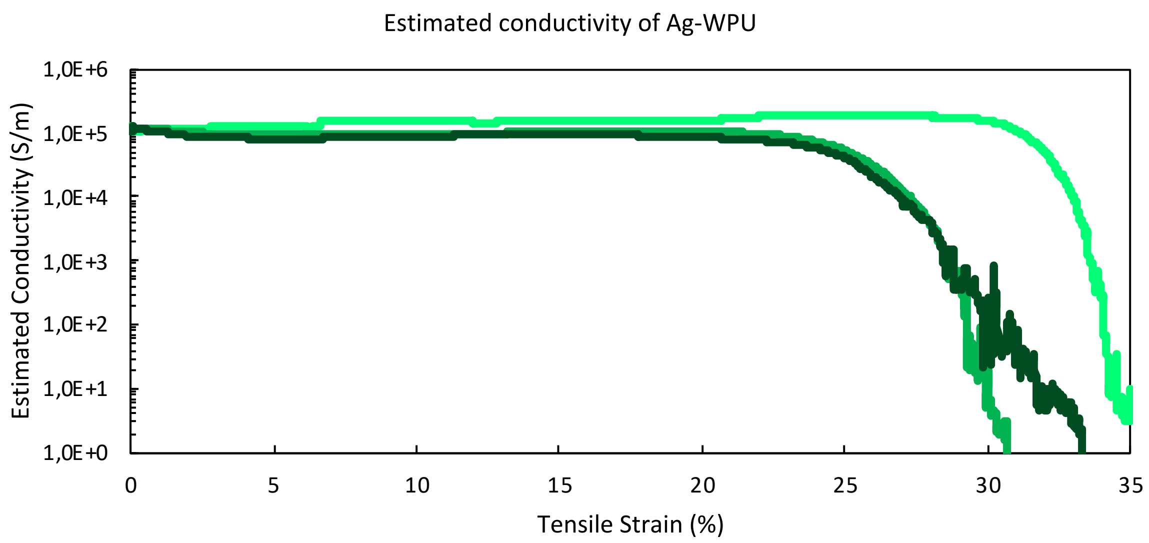

Figure S4. Estimated conductivity of Ag-WPU samples upon stretching. The material was assumed to be incompressible.

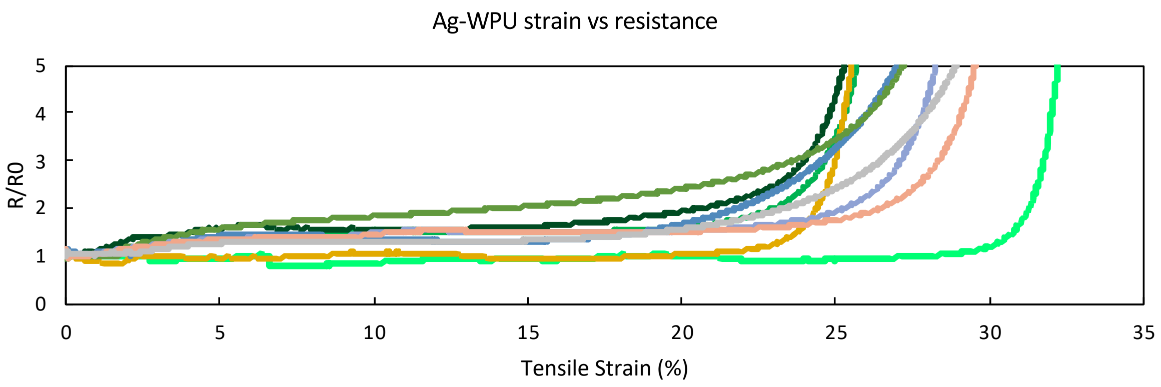

Figure S5. Strain vs resistance curve for Ag-WPU traces printed over a thermoplastic polyurethane (TPU) substrate (nine samples from distinct ink batches).

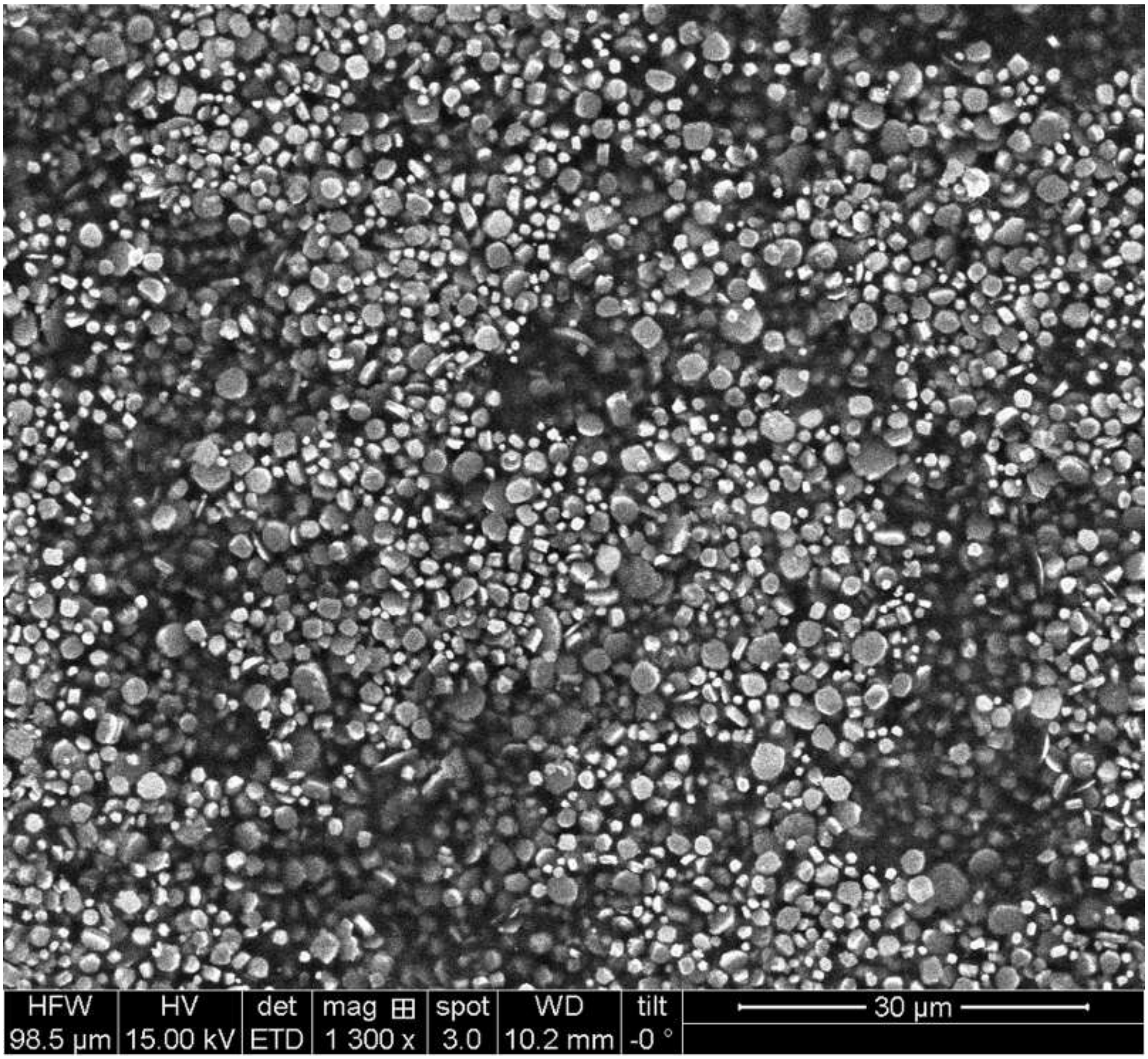

Figure S6. SEM image of Ag-WPU surface

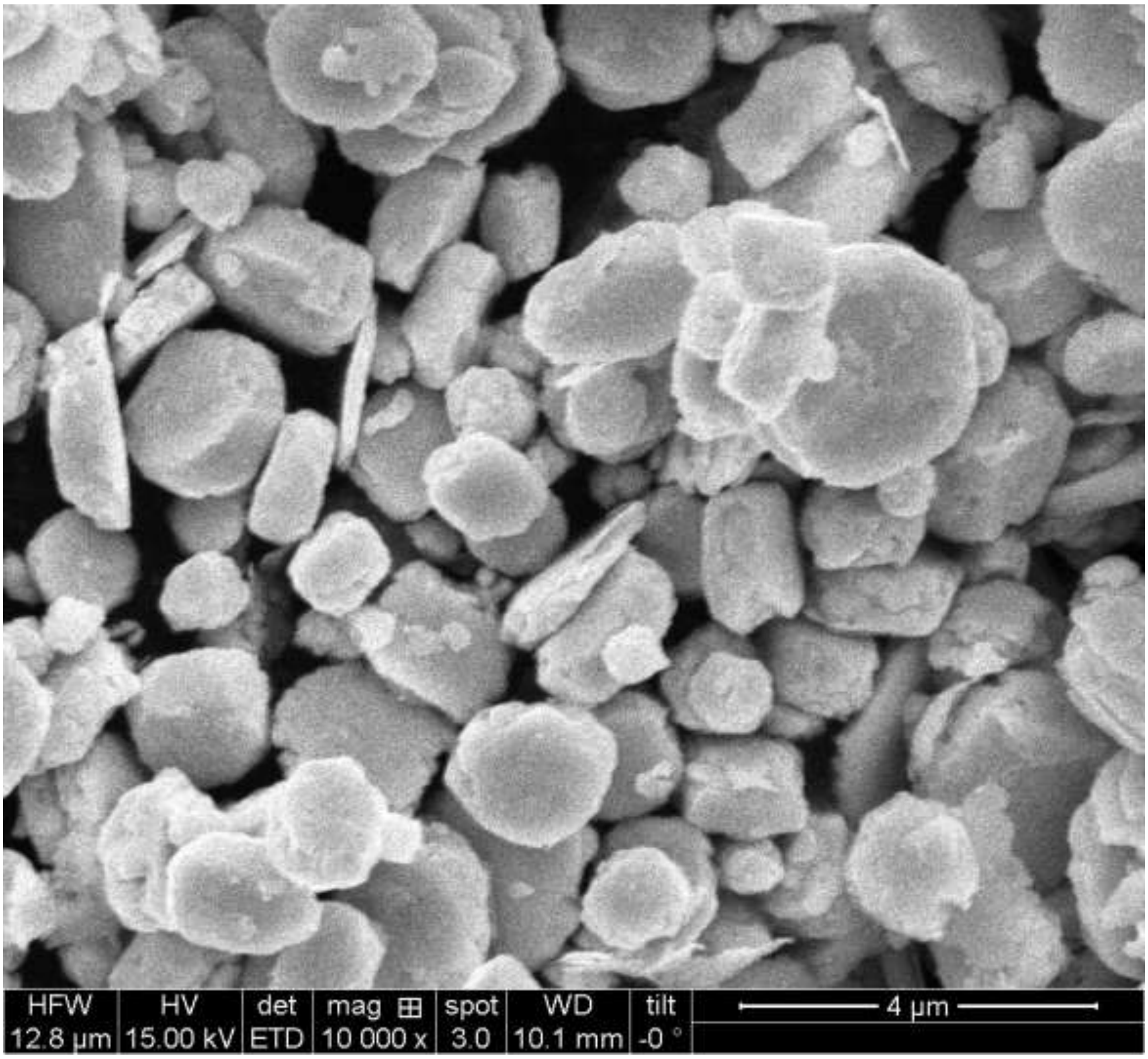

Figure S7. SEM image of Ag-WPU surface

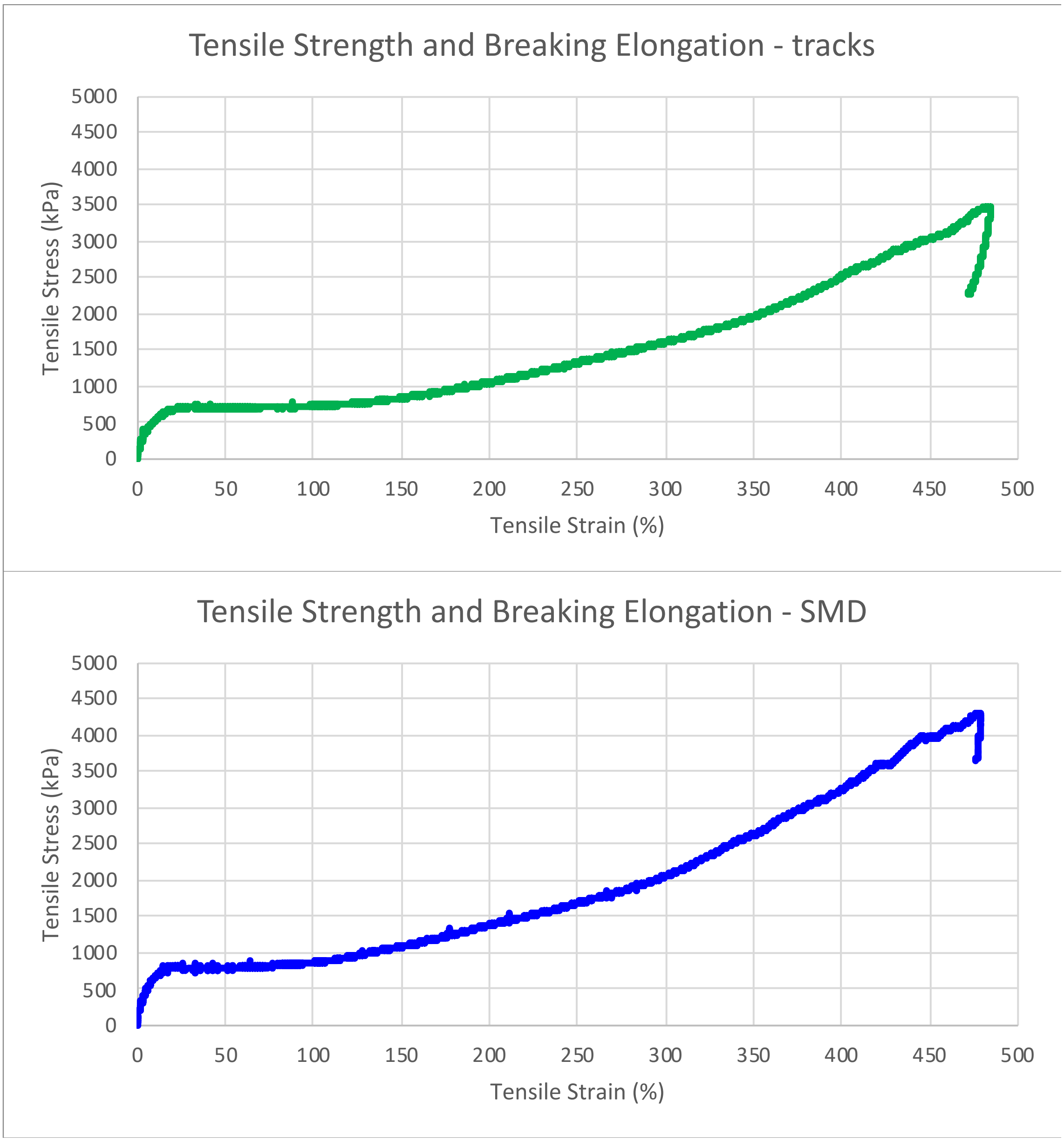

Figure S8. Strain-Stress curves until break for printed ink trace (green) and printed trace with integrated SMD resistor (blue).

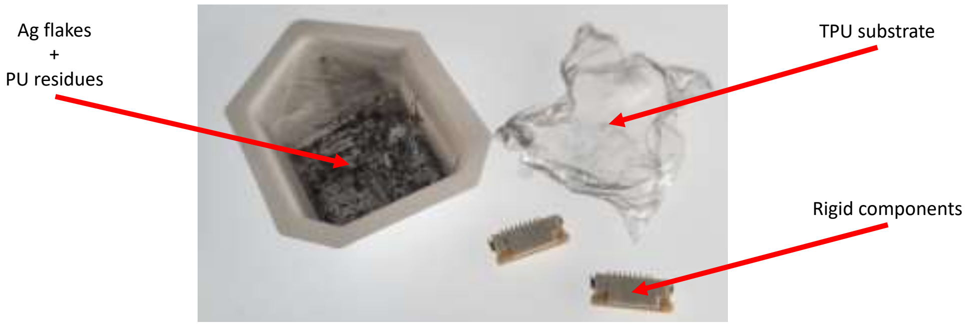

Figure S9. Printed circuit with integrated SMD components separated in its fundamental elements: conductive ink, TPU substrate, and rigid components.

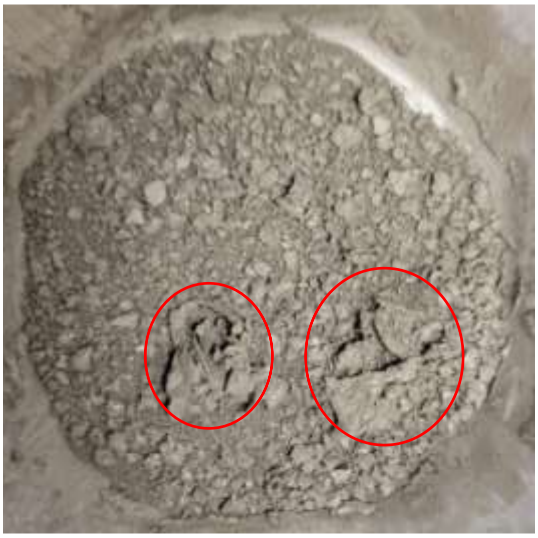

Figure S10. Ag-rich powder after IPA washing. Large PU aggregates can be observed (red highlights).

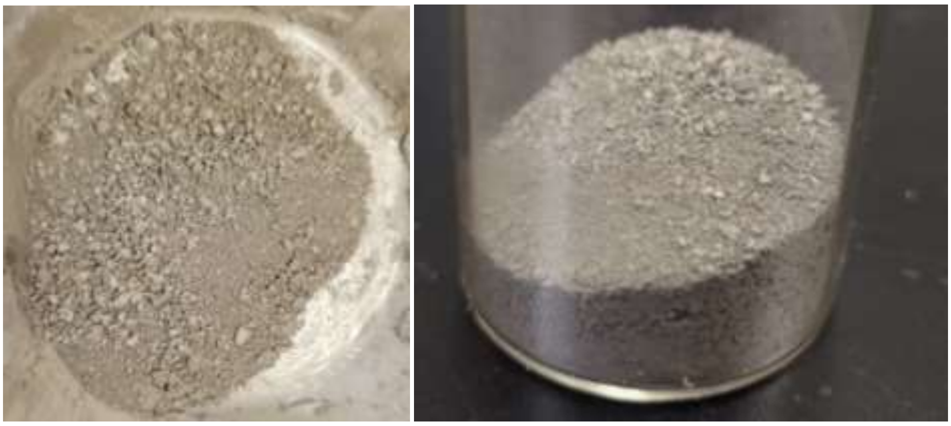

Figure S11. Ag-rich powder after removal of large PU chunks (left) and after being transferred to a glass vial (right).

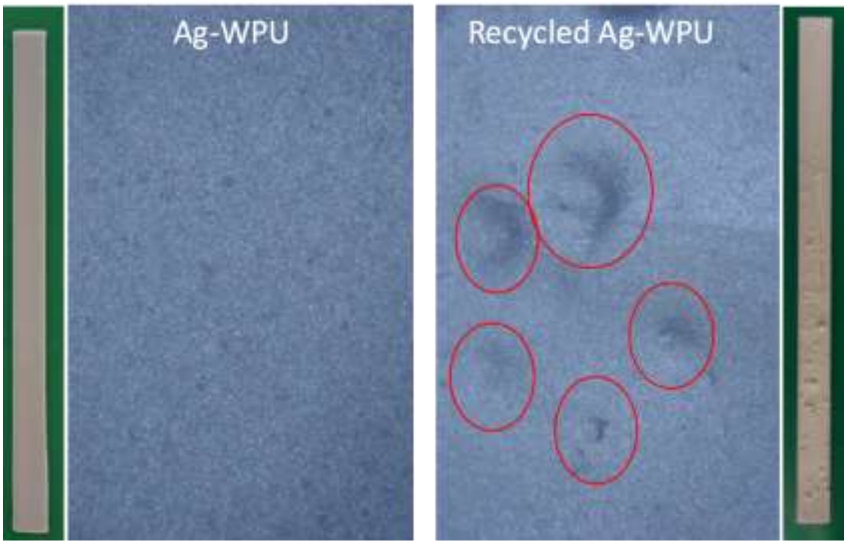

Figure S12. Microscope images of the surface of printed traces from pristine Ag-WPU ink (left) and recycled Ag-WPU ink (right). For the image on the right, a rough surface can be observed, originating from solidified PU residue trapped in the print.

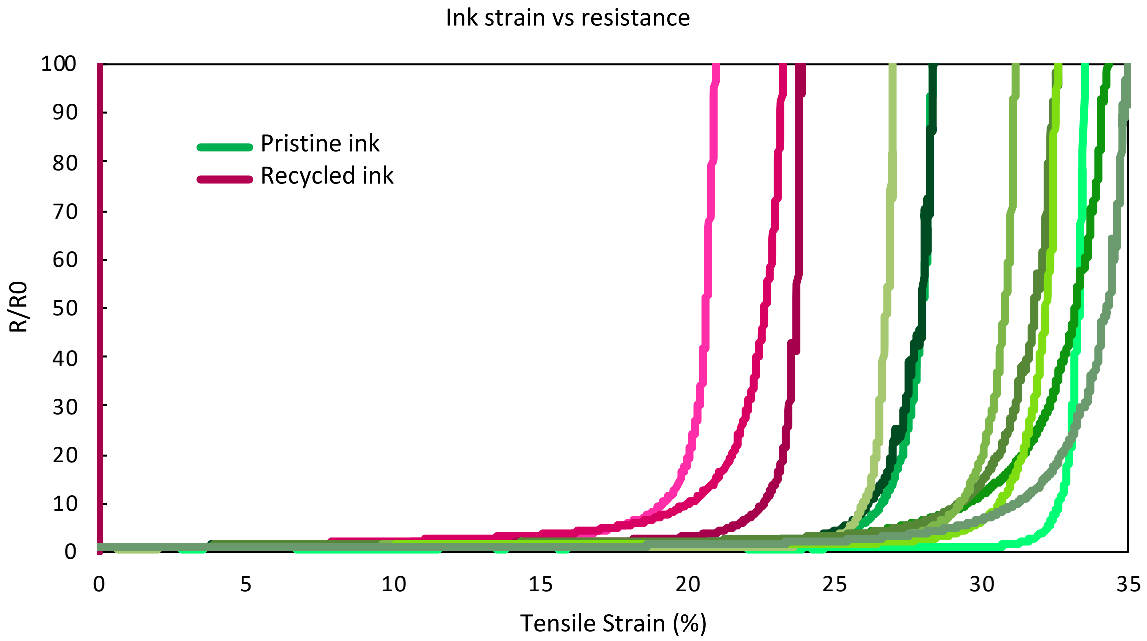

Figure S13. Comparison between Strain vs resistance curve for pristine Ag-WPU traces and traces printed from recycled Ag-WPU.

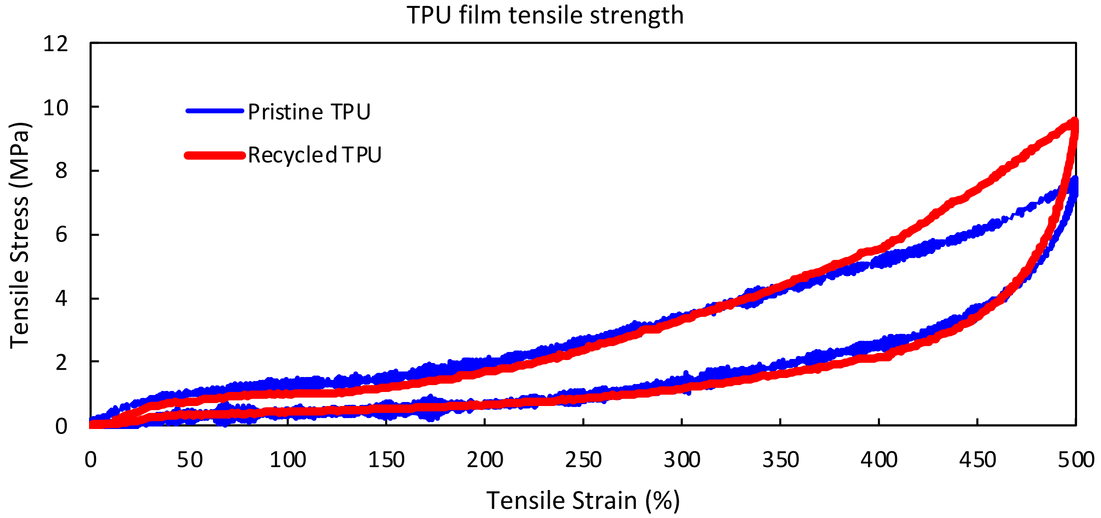

Figure S14. Strain-stress curves for pristine TPU film and the same TPU film after undergoing the IPA recycling process.

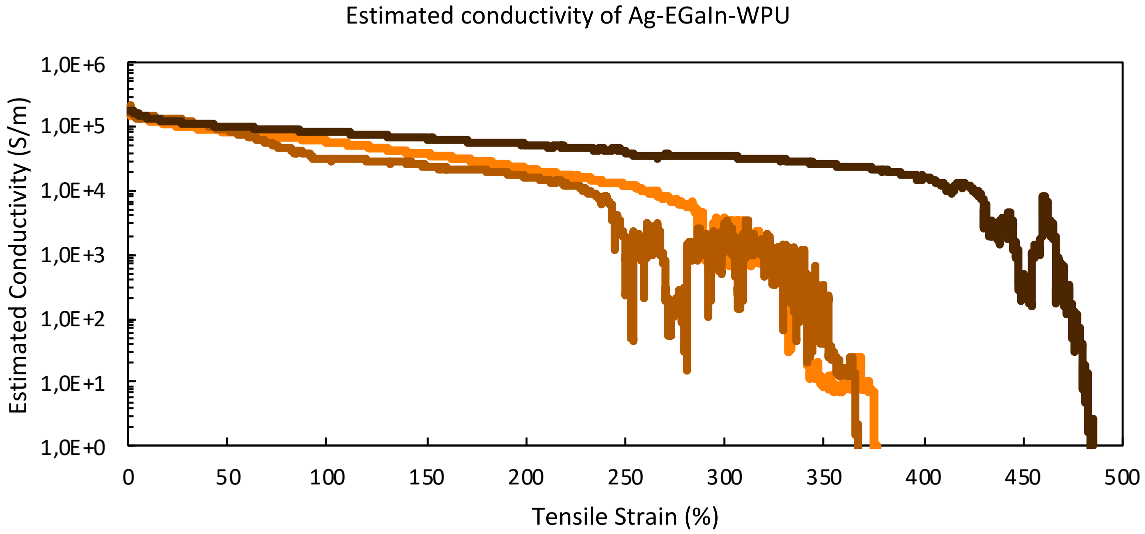

Figure S15. Estimated conductivity of Ag-EGaIn-WPU samples upon stretching. The material was assumed to be incompressible.

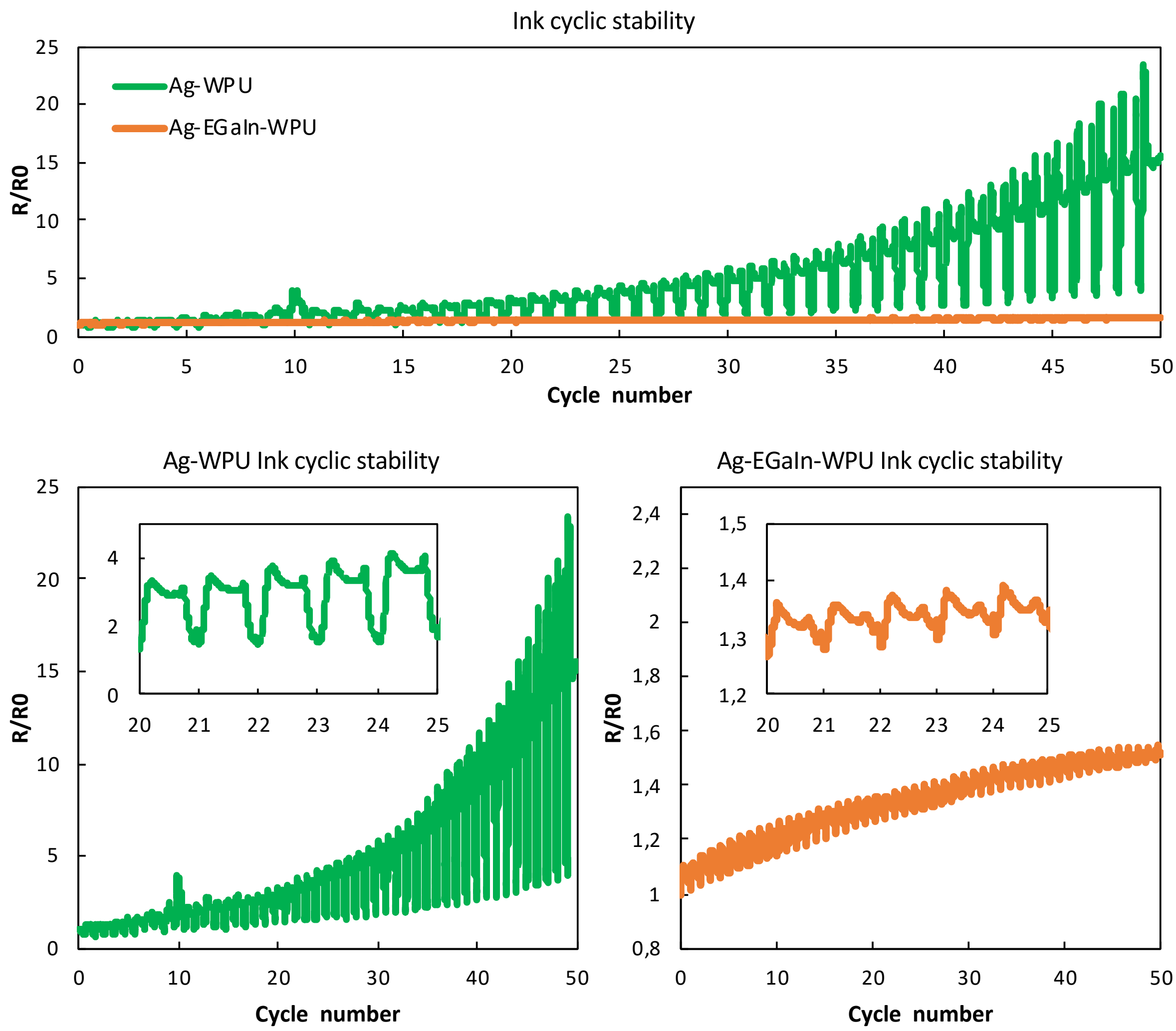

Figure S16. 50 stretching/releasing cycle test of the Ag-WPU and Ag-EGaIn-WPU inks (ε = 10%). The insets on the bottom plots show 5 cycles in the middle of the test.

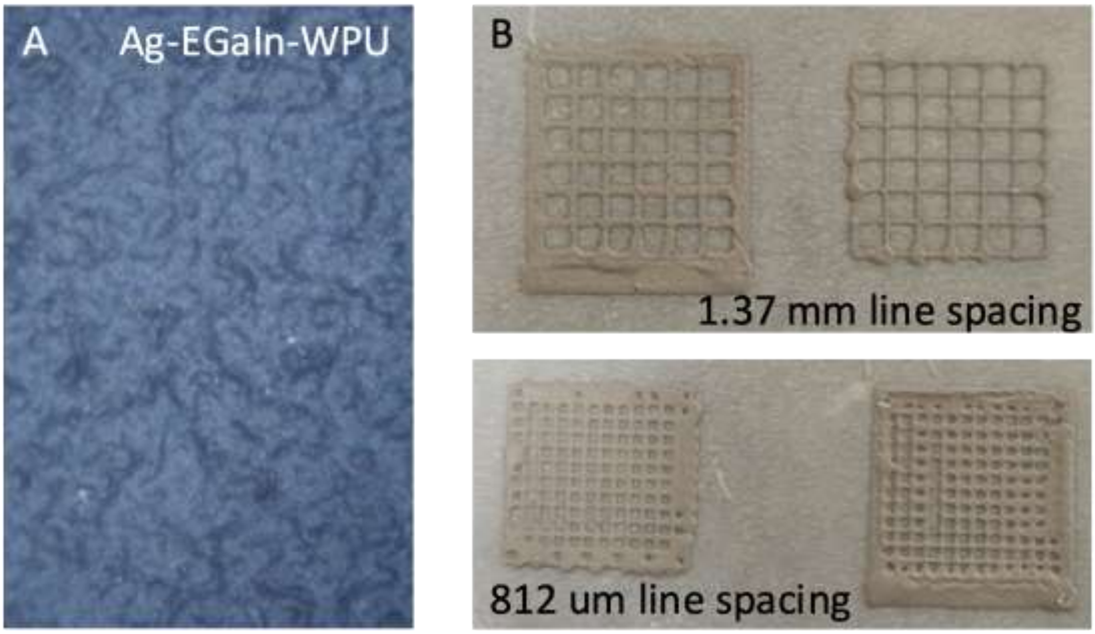

Figure S17. A: Microscope images of the surface of printed traces from Ag-EGaIn-WPU. A rough surface can be observed, originating from EGaIn droplets fully encapsulated in polyurethane. **B:** DIW printed grids using the Ag-EGaIn-WPU ink with 1.37 mm and 812 um line spacing. A 250 um diameter nozzle was used.

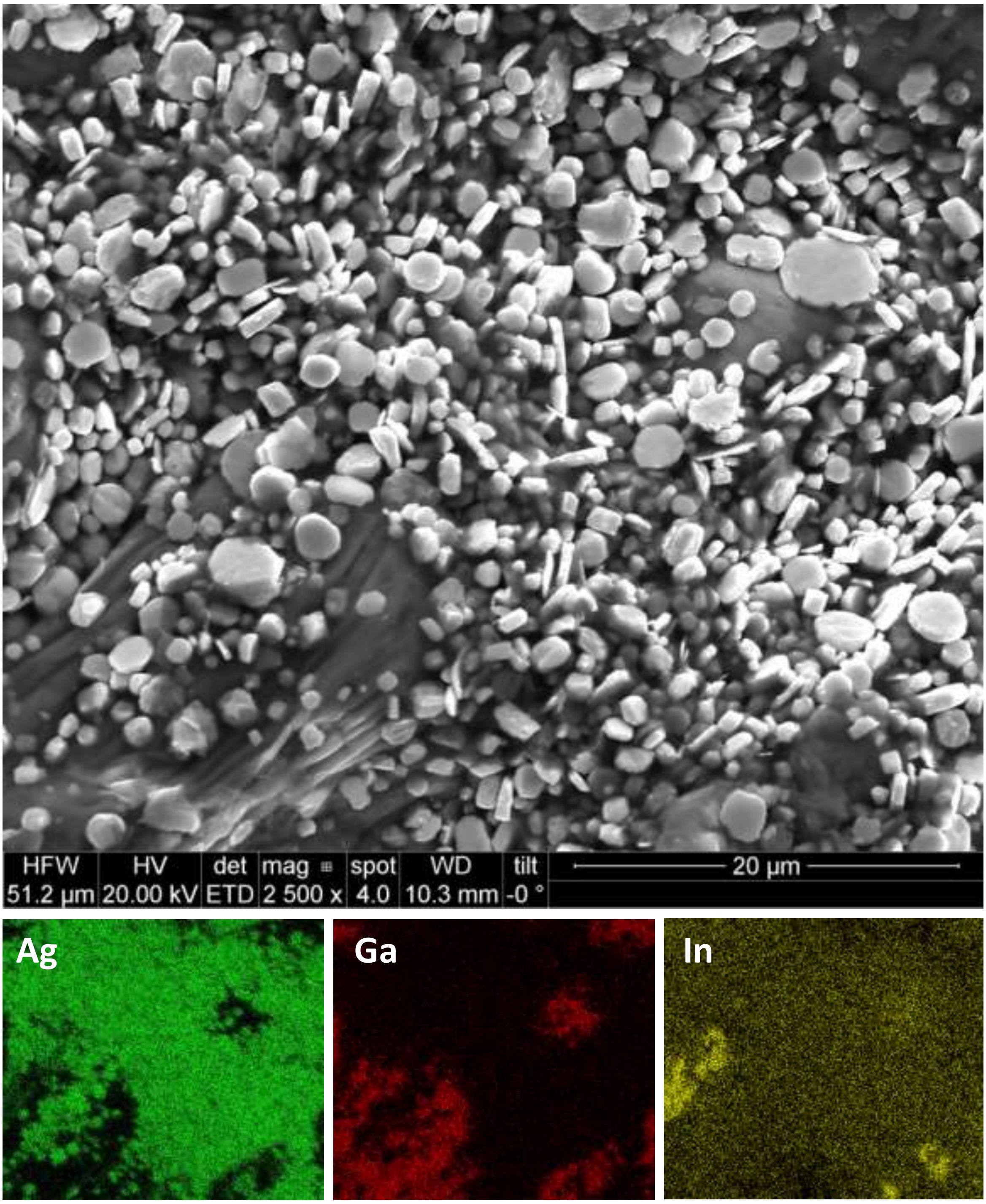

Figure S18. SEM image and EDS analysis of Ag-EGaIn-WPU sample cross section. A clear separation between Ag and Ga can be observed, while In is present all over the sample

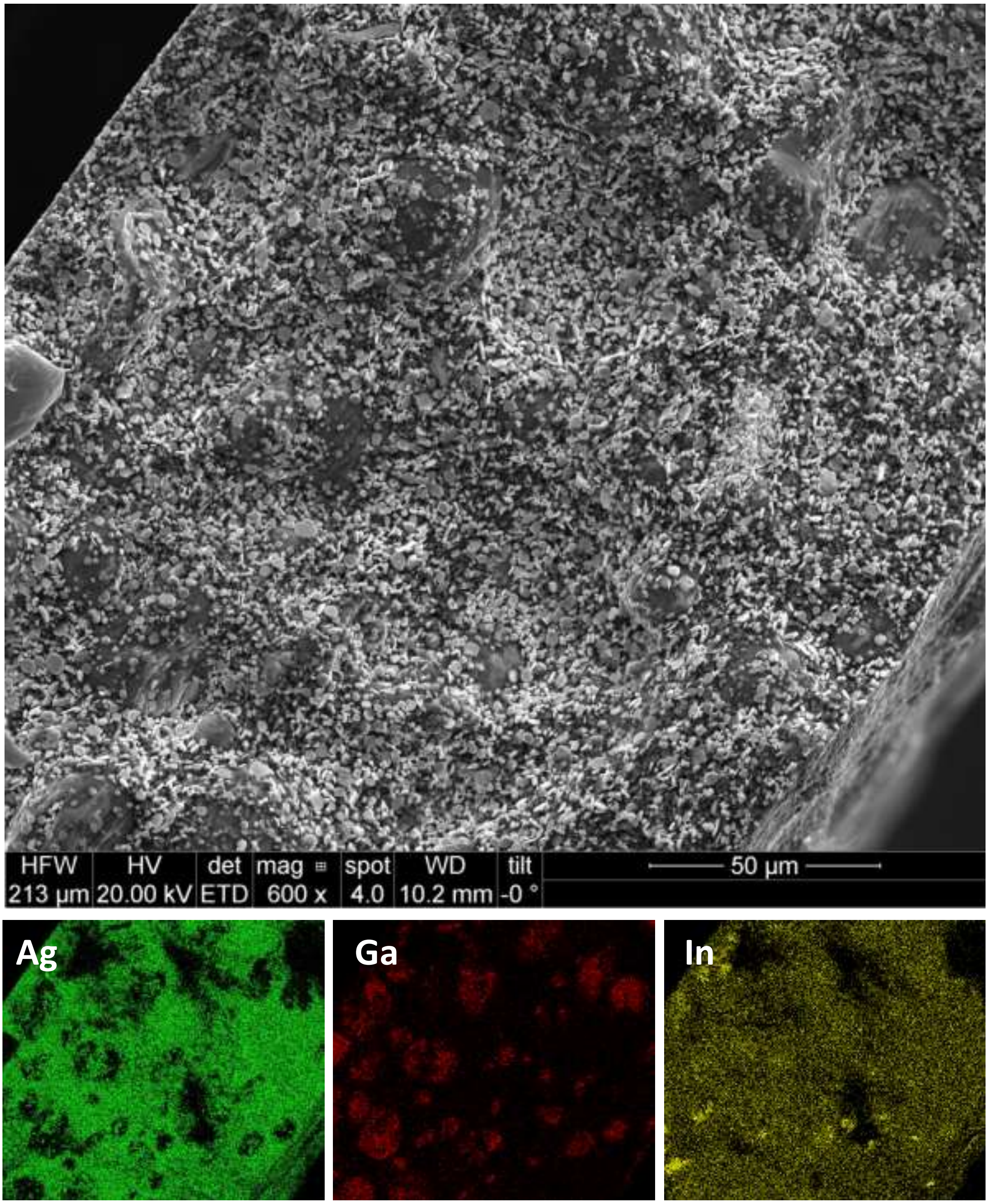

Figure S19. SEM image and EDS analysis of Ag-EGaIn-WPU sample cross section. A clear separation between Ag and Ga can be observed, while In is present all over the sample

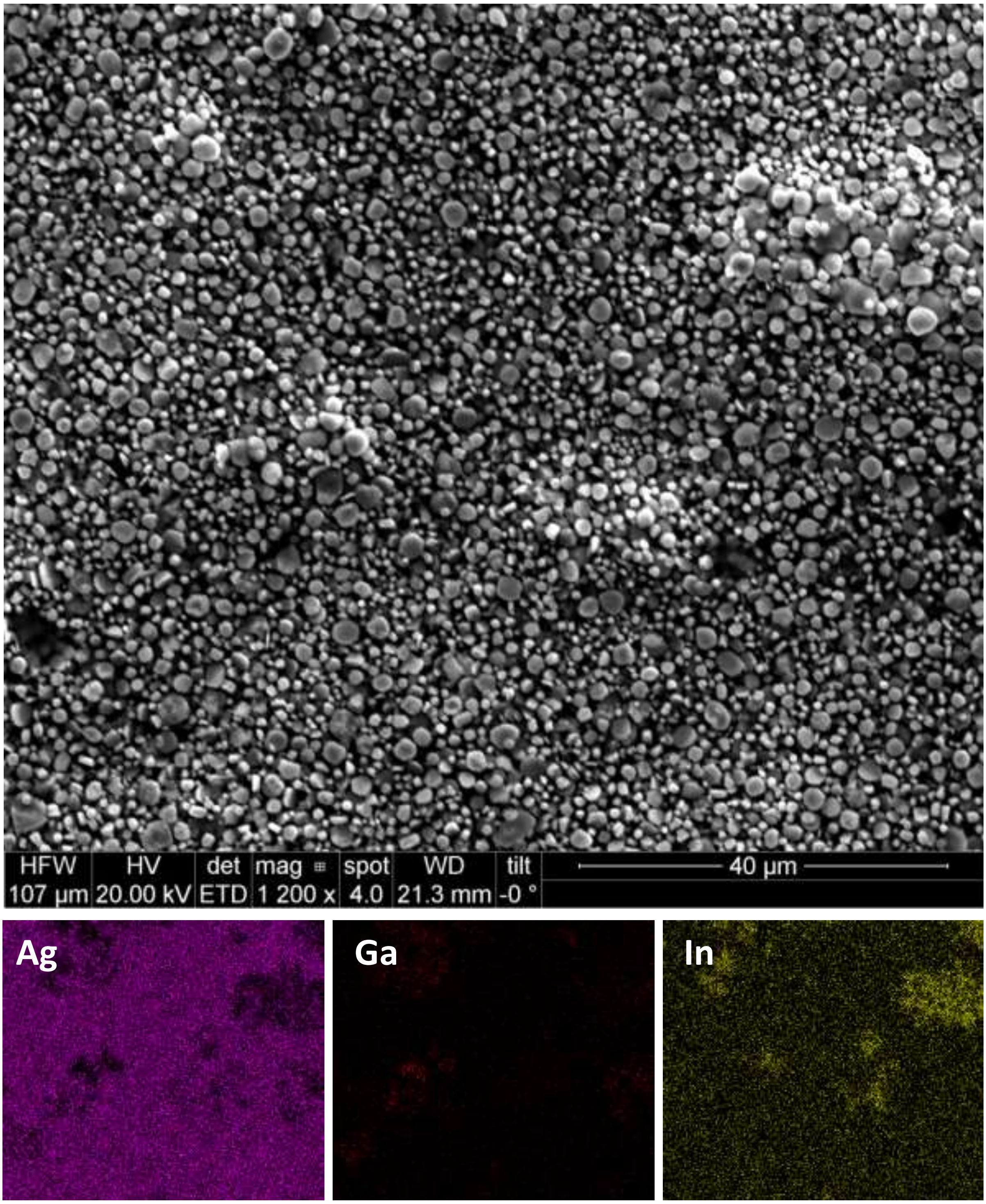

Figure S20. SEM image and EDS analysis of Ag-EGaIn-WPU sample cross section. The formation of intermetallic Ag-In microparticles is observed. These can be distinguished from silver flakes thanks to their spherical shape

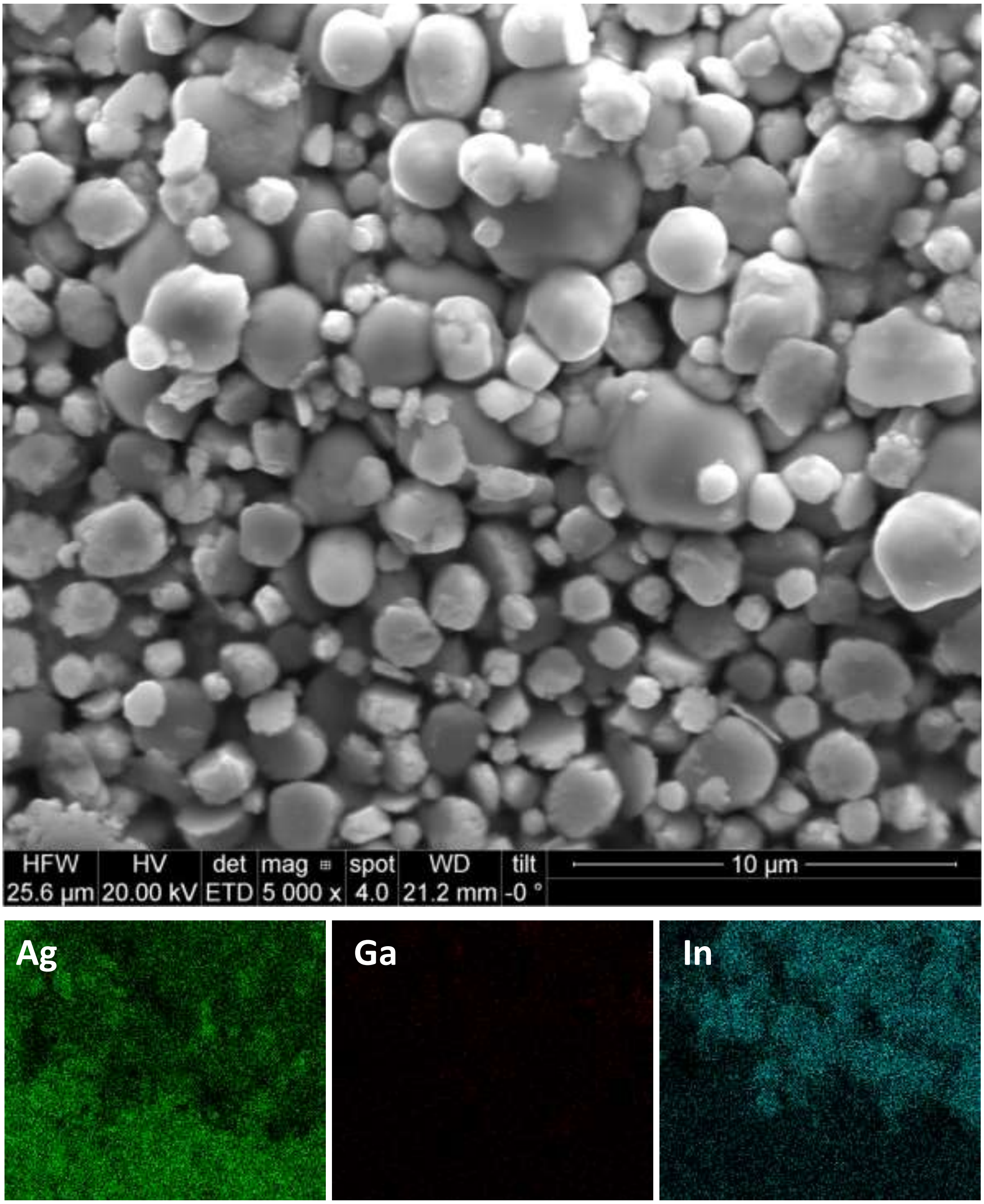

Figure S21. SEM image and EDS analysis of Ag-EGaIn-WPU sample cross section. The formation of intermetallic Ag-In microparticles is observed. These can be distinguished from silver flakes thanks to their spherical shape

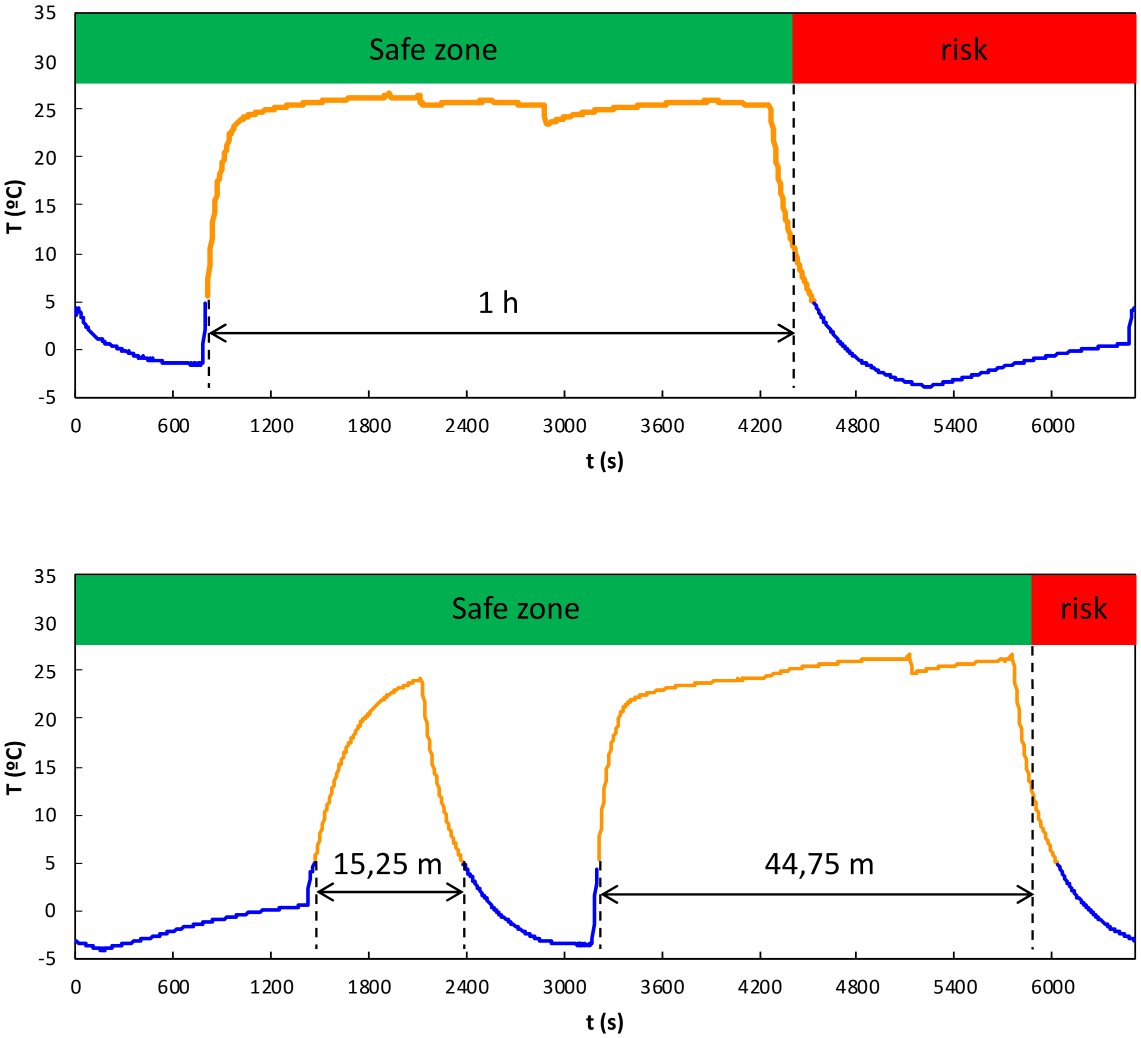

Figure S22. Temperature plots from data acquired by the printed smart label. The safe zone (green LED) occurs while the packages are not stored above 5 ºC for more than 1 hour. When this 1 hour time limit is passed, the risk zone (red LED) is entered, even if the package is further correctly refrigerated (below 5 ºC). the 1 hour limit is cumulative, as shown in the bottom plot.

Smiling

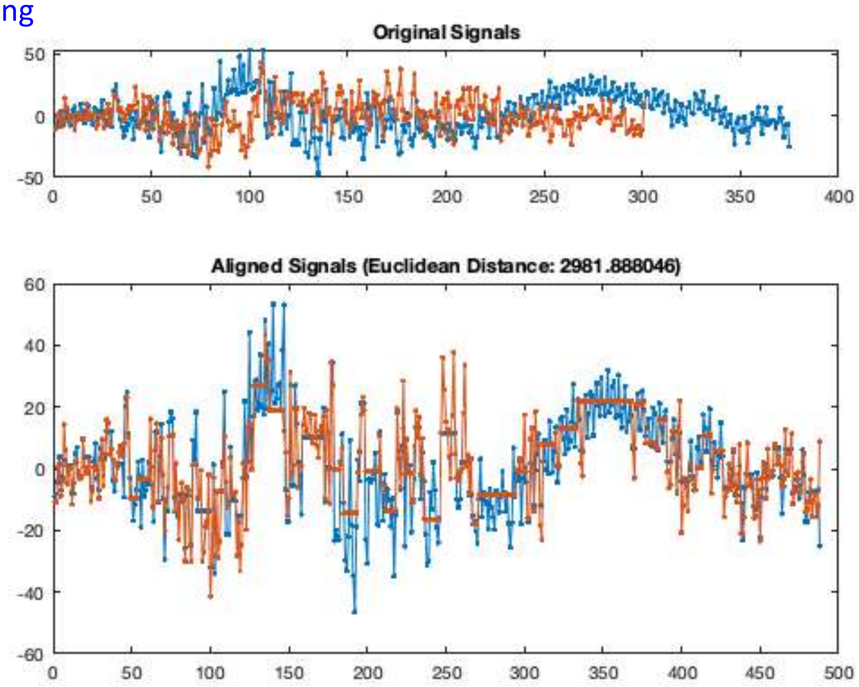

Figure S23. Dynamic time warping fitting between the signals for two repetitions of a smiling gesture.

Jaw clenching

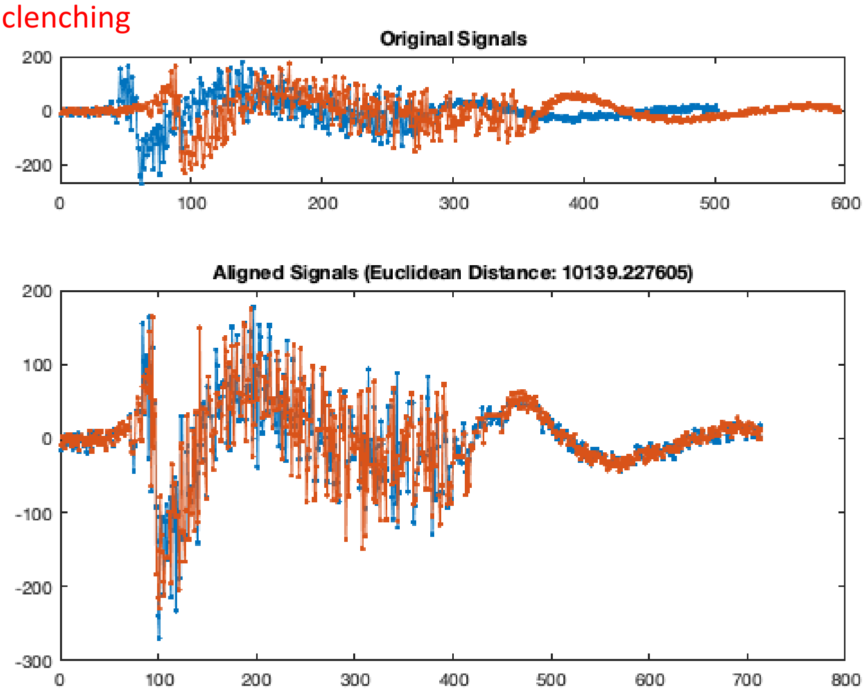

Figure S24. Dynamic time warping fitting between the signals for two repetitions of a jaw clenching gesture.

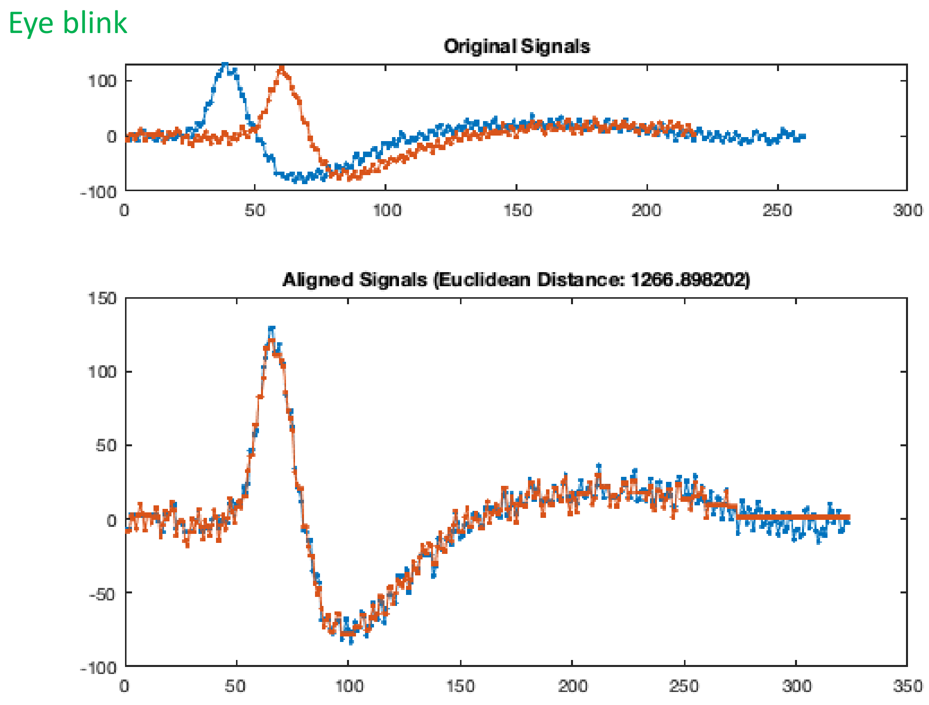

Figure S25. Dynamic time warping fitting between the signals for two repetitions of an eye blinking gesture.

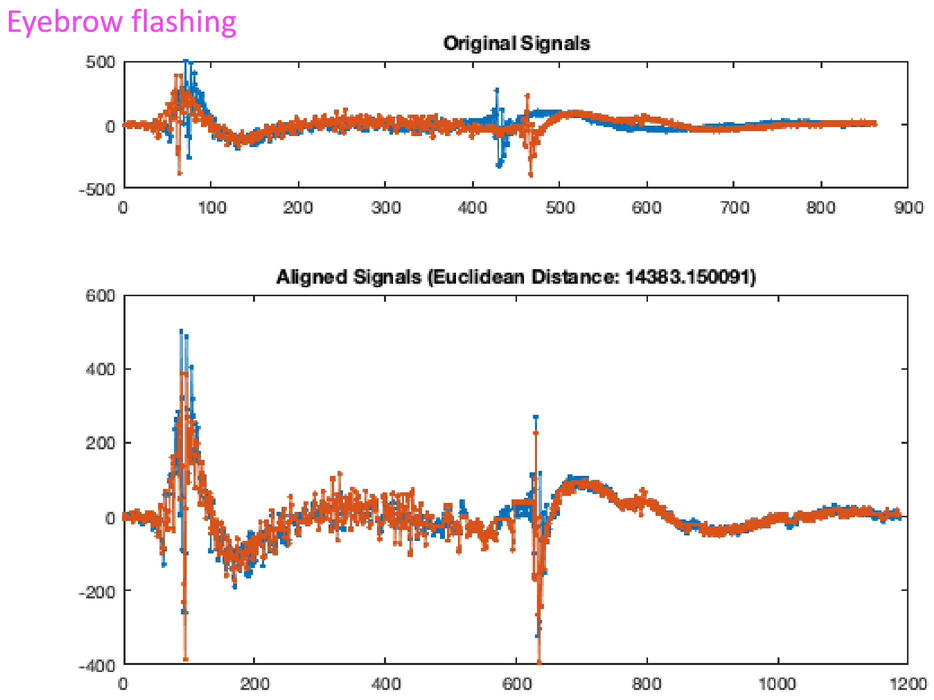

Figure S26. Dynamic time warping fitting between the signals for two repetitions of an eyebrow flashing gesture.

		1st repetition			
		smiling	jaw clenching	eye blinking	eyebrow flashing
2nd repetition	smiling	2981,888	12808,457	4096,79	23929,906
	jaw clenching	15103,017	10139,228	16960,927	20410,614
	eye blinking	3589,393	13914,421	1266,898	23090,905
	eyebrow flashing	23008,425	17209,825	22210,972	14383,15

Figure S27. Euclidean distance between gesture pairs of two repetitions, obtained from Dynamic Time Warping of the electrophysiological signals.

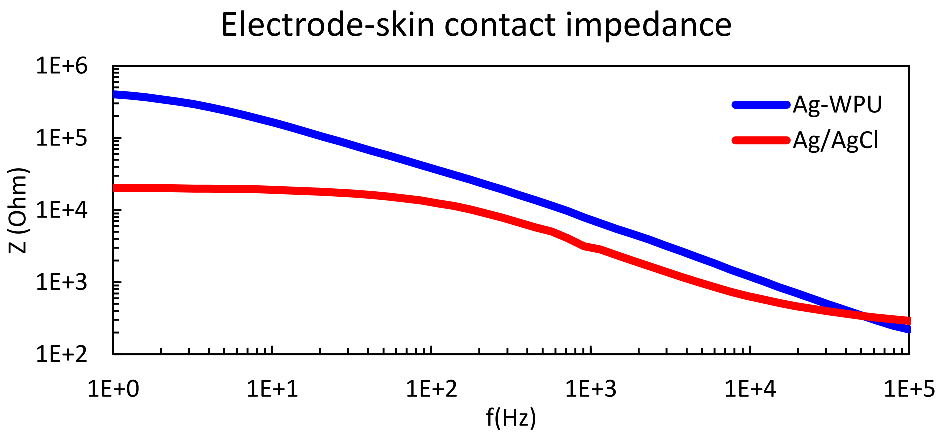

Figure S28. Electrode-skin impedance (1 Hz-500 kHz) for printed Ag-WPU electrodes and medical-grade Ag/AgCl electrodes.

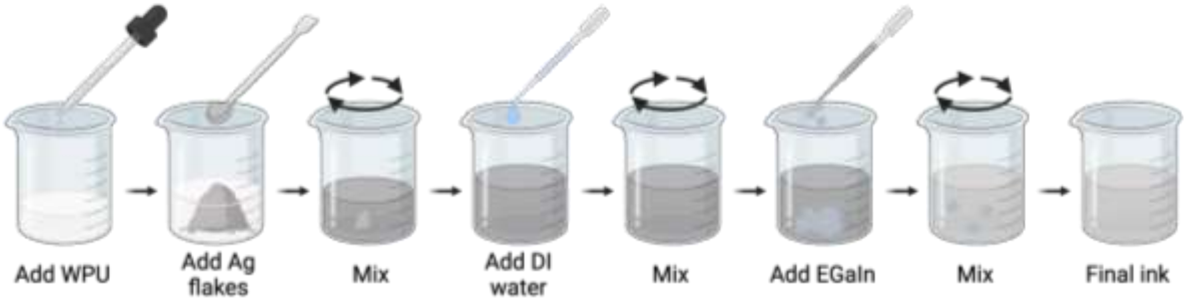

Figure S29. Steps for synthesis of Ag-EGaIn-WPU conductive ink. An overhead stirrer was used instead of a planetary mixer.

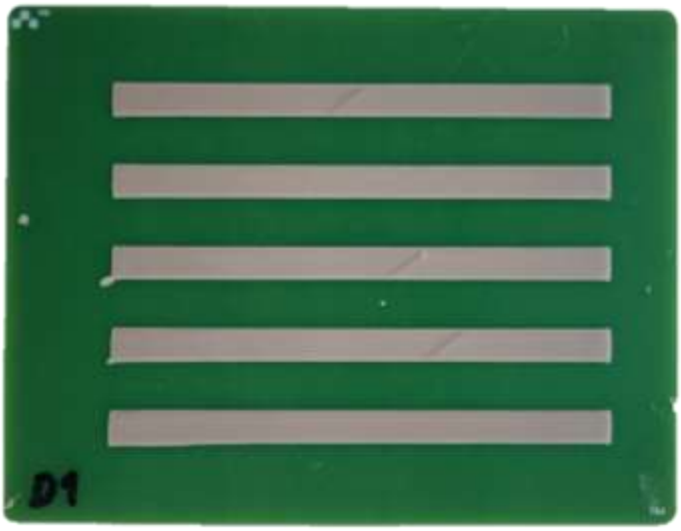

Figure S30. Example of the printed traces over FR1 substrate to measure ink conductivity.

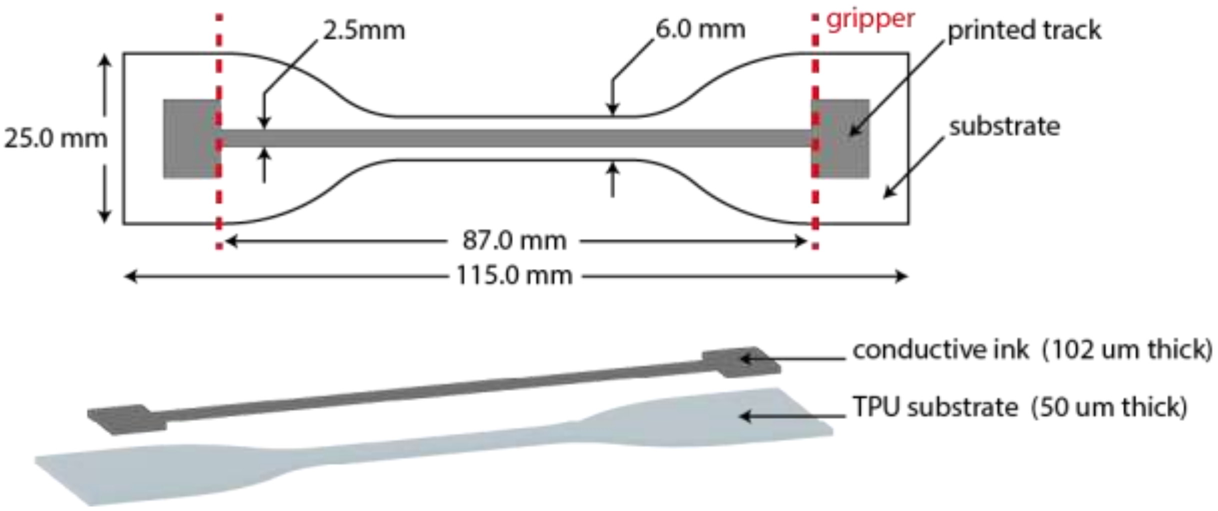

Figure S31. Die C, ASTM D412 dogbone with printed trace for tensile testing.

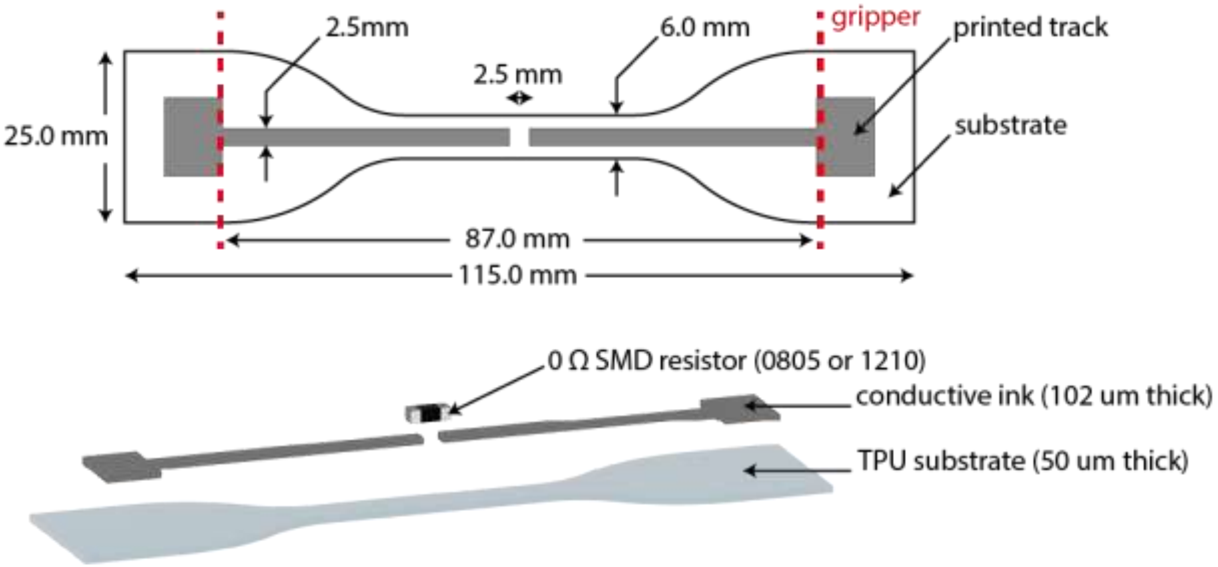

Figure S32. Die C, ASTM D412 dogbone with printed trace and integrated SMD resistor for tensile testing.

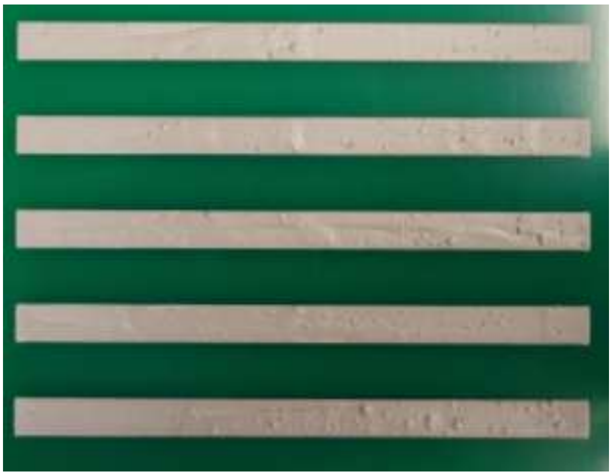

Figure S33. Example of the printed traces over FR1 substrate using recycled Ag-WPU ink. Rough surface texture arises from the presence of cured polyurethane particles in the recovered Ag-rich powder that become encapsulated in the new print.

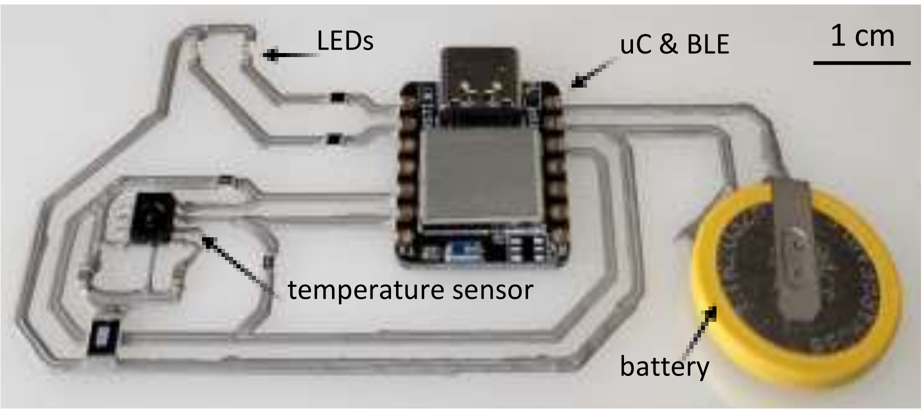

Figure S34. Recyclable smart label circuit.

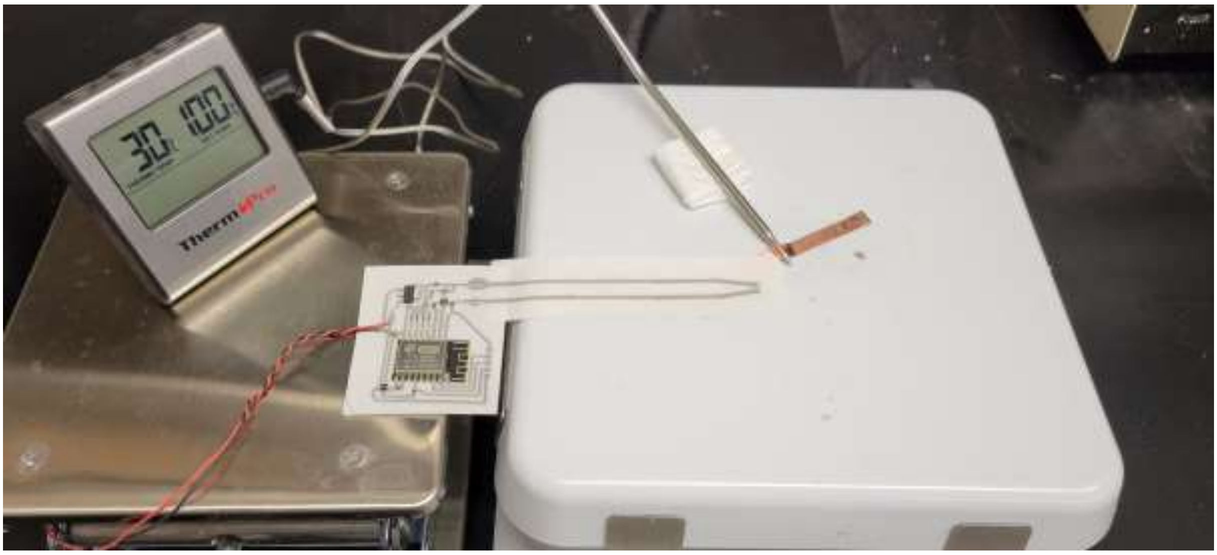

Figure S35. Setup for thermistor calibration consisting of a hotplate and digital thermometer.

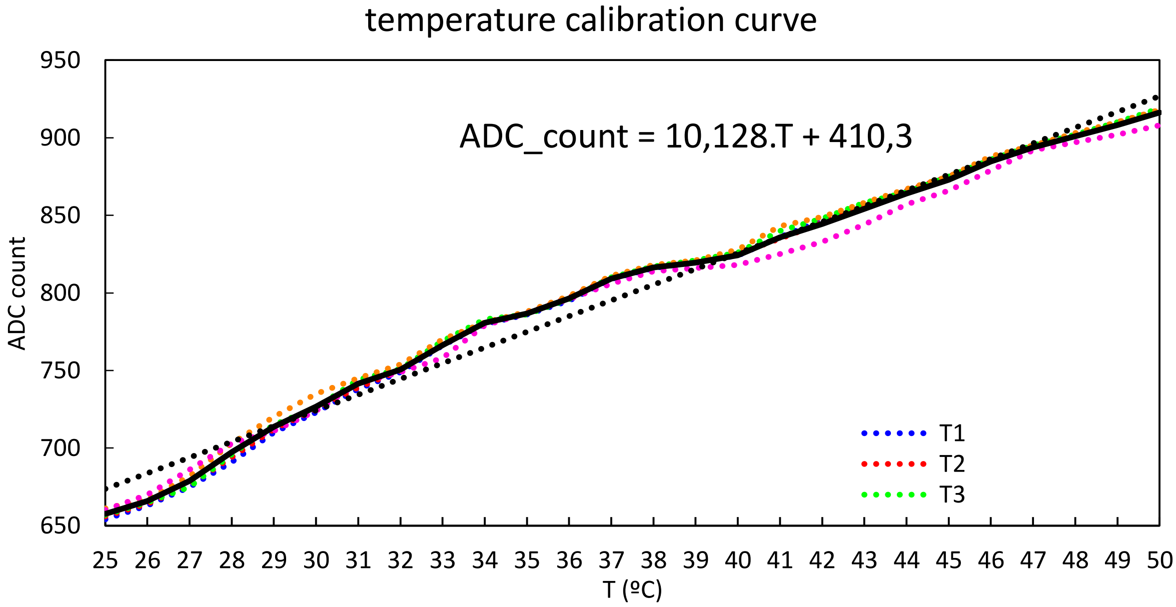

Figure S36. Calibration curve for the thermistor-based temperature-monitoring electronic patch.

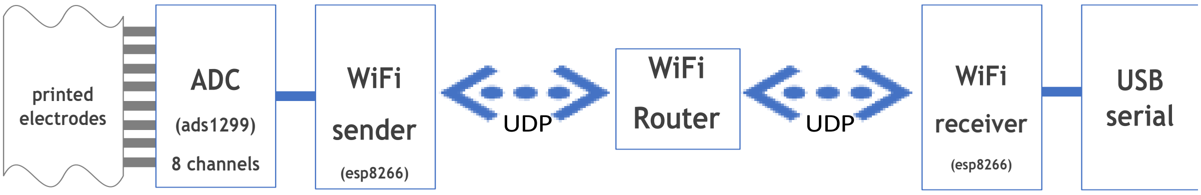

Figure S37. High level block diagram of the developed electrophysiology recording system.
